# Relation between colour- and phase changes of a leuco dye-based thermochromic composite

**DOI:** 10.1038/s41598-018-23789-2

**Published:** 2018-04-03

**Authors:** Kristina Bašnec, Lidija Slemenik Perše, Boštjan Šumiga, Miroslav Huskić, Anton Meden, Aleš Hladnik, Bojana Boh Podgornik, Marta Klanjšek Gunde

**Affiliations:** 1 Radeče Papir Nova, d.o.o., Njivice 7, SI-1433 Radeče, Slovenia; 2 National Institute of Chemistry, Hajdrihova 19, SI-1000 Ljubljana, Slovenia; 3 University of Ljubljana, Faculty of Natural Sciences and Engineering, Aškerčeva cesta 12, SI-1000 Ljubljana, Slovenia; 4 University of Ljubljana, Faculty of Chemistry and Chemical Technology, Večna pot 113, SI-1000 Ljubljana, Slovenia; 5 Present Address: University of Ljubljana, Faculty of Mechanical Engineering, Aškerčeva 6, SI-1000 Ljubljana, Slovenia

## Abstract

Reversible colour change of leuco dye-based composites is in general closely related to their phase change, thus the two phenomena should occur at around the same temperature and should be influenced similarly. However, spatial confinement of the analysed sample affects the change in colour differently compared to its phase transition and the most pronounced effects can be observed during cooling. The bulk composite is coloured while still liquid and the colour hysteresis does not exhibit a loop. In an open-porous medium the colouration coincides well with the crystallization and the colour hysteresis widens to about 4 °C. Microencapsulated composite exhibits two crystallization processes, one of them taking place at the bulk crystallization temperature and the other one at about 20 °C lower. Under such conditions the composite is coloured just before the onset of the second crystallization, i.e. about 15 °C below crystallization in the bulk, and the corresponding colour hysteresis widens to 18 °C. The two crystallization forms are thermally independent and have the same crystalline structure. These effects should be taken into account when designing future applications where the phase-changing materials are implemented.

## Introduction

Organic thermochromic (TC) materials are applied in a wide range of commercial products such as in colour-changing textiles^1–4^, for temperature control of a foldable paper or textile^5^ and even for colour-changing wood veneers^6^. In these applications the TC material is used in a spatially confined form. Most frequently, microencapsulation is used to produce colour-changing “pigments” suitable for printing inks.

During the last decades, the leuco dye-based TC materials have received substantial attention from both researchers and industry. In these materials, the temperature-induced colour change occurs due to molecular rearrangement of colour formers (leuco dyes) in the presence of a developer and this process is controlled by a phase change of the co-solvent. The molecular mechanisms were thoroughly studied in fully coloured as well as in totally decoloured states, i.e. in solid and liquid states of the composite, respectively^7–14^. However, a leuco dye-based TC composite can solidify without changing its colour, and the colourless solid state can exist within a reasonably wide temperature region^15^. Such a state has not yet been studied in more detail.

The colour change as a function of temperature produces a hysteresis which is very narrow for TC composites and much wider for printing inks where these are microencapsulated to protect the phase-changing material (PCM) from its surrounding^16–19^. The shape of the colour hysteresis provides the first evidence of changes in the TC composite, such as poor fastness against light and high temperatures^18^. To the best of our knowledge, the relationship between the phase- and colour changes has not yet been analysed.

The phase changes of leuco dye-based TC composites are controlled by the applied co-solvent, which can be a long-chain alkyl alcohol, ester, ketone, ether, or acid^1,2^. Such PCMs were extensively investigated to determine the relationship between their structure and energy storage properties in eutectic mixtures for the solid-liquid transition^20–24^. PCMs are commonly used in a microencapsulated form^1,25–30^ but this can change their thermal properties when measured in the bulk^31^. In many cases, supercooling lowers crystallization temperature, depending on the type of polymer shell and the size of microcapsules^32–35^. A greatly reduced number of nucleation sites induces an independent crystallization within isolated parts of the material, known as homogeneous nucleation. Different nucleation efficiency in microcapsules of various sizes may result in multiple crystallization peaks^31^. Supercooling of 10 °C and more was investigated during the crystallization of mini-emulsion droplets (immiscible liquid drops of 100–500 nm) of alkanes^36,37^. Mini-emulsions of immiscible polymer blends with a broad size distribution of droplets were shown to have multiple crystallization peaks; a broader size distribution leads to a larger number of peaks^38^.

Large effects were also observed in porous media. Crystallization of a fluid in a nanometer-sized porous material resulted in a much lower freezing temperature, i.e. in a more pronounced supercooling effect, which was described as a layer-by-layer crystal growth^39^. Freezing of n-alcohols in mesoporous silicon was obtained at 28 °C lower temperature than in the bulk^40^.

The most important research of a TC composite in spatially confined conditions was done for its application as the active core in fibres made by melt coaxial electrospinning. It was shown that the thermal and colouration properties of the active core were only slightly affected in fibres with a diameter of 0.5–2 µm^3^, but many changes were found in fibres with a diameter of 1.7–5.7 µm^4^. Multiple solidification peaks observed in dynamic DSC curves were contributed to different diameters of the fibre’s core and the change in freezing temperature was linked to possible reactions between the polymer shell and the active core inside the microcapsules. It was also concluded that the phase transition temperature might not be the only parameter governing the colour transition temperature^4^.

The above results were a strong motivation for our research. If the colour of leuco dye-based TC composites follows their phase transition temperatures, their colour and phase changes should be equally affected by a spatial micro-arrangement. To analyse this assumption, the same TC composite was studied both in the bulk and in a confined form. Because TC composites can only seldom be applied in the bulk, the obtained results should be important for a design of the functional properties of the final application.

## Results and Discussion

### Colour properties

The shape of the hysteresis was described by the loop width and four characteristic temperatures, describing the onset (T_1_ and T_3_) and termination (T_2_ and T_4_) of the TC effect: discolouration at heating (T_1_ and T_2_) and colouration at cooling (T_3_ and T_4_), respectively (Fig. S1, Supplementary information).

**Figure 1:**
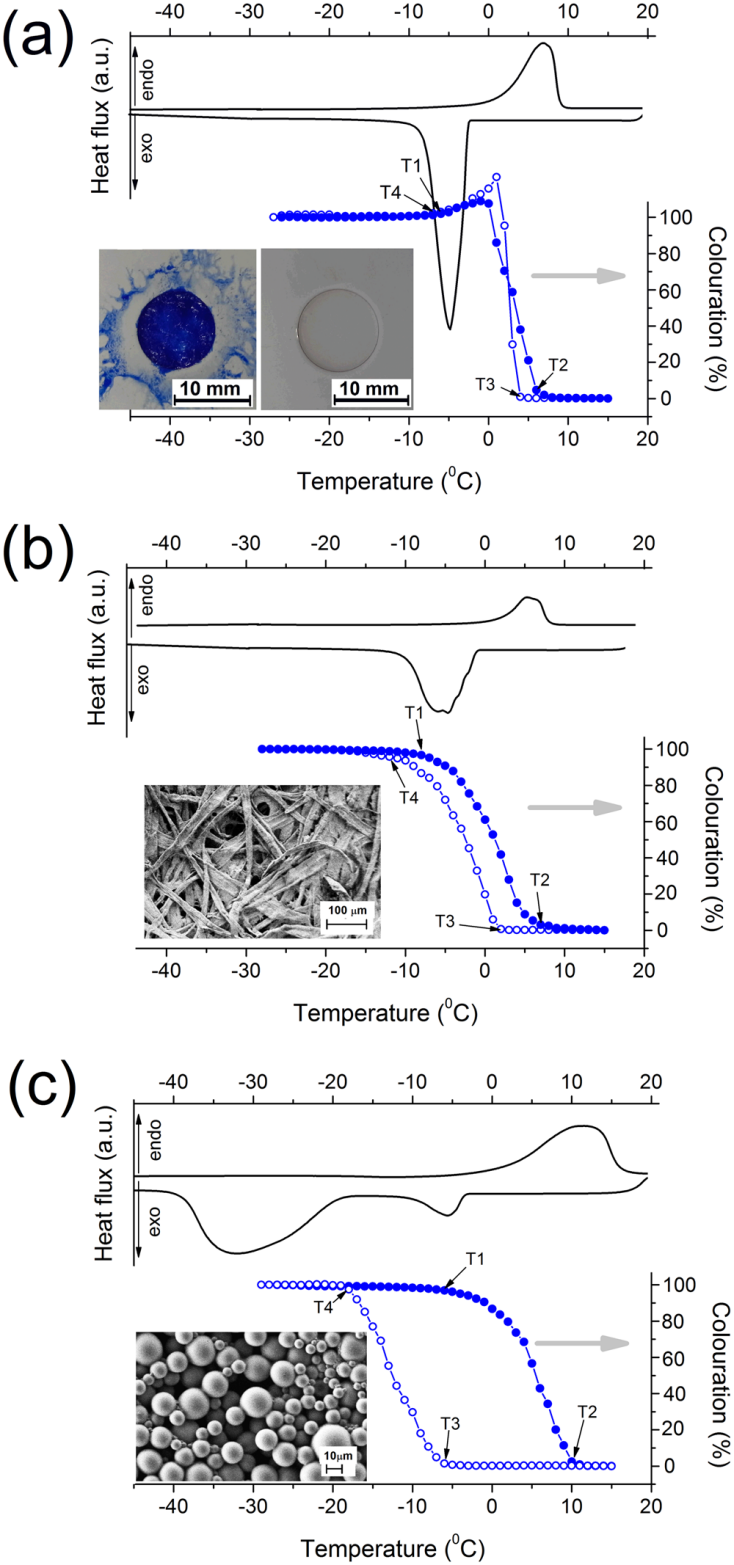
Thermal (DSC, black curve) and colorimetric properties (blue curve with circles) of the (**a**) TC composite in the bulk, (**b**) inside the chromatographic paper and (**c**) in microcapsules. Heating and cooling rates of 5 °C/min and 10 °C/min, respectively, were used for both measurements. Pictures of the corresponding holders are included. The bulk composite is shown in the fully coloured and totally discoloured states (**a**).

The temperature-dependent colour and thermal properties of the three forms of the TC composite are shown in Fig. 1 and the data describing the colour hysteresis in Table 1. The same heating and cooling rates (5 °C/min and 10 °C/min, respectively) were used to enable comparison between the two measurements.

**Table 1:** The onset (T_1_ and T_3_) and termination (T_2_ and T_4_) temperatures of the TC effect - discolouration at heating (T_1_ and T_2_) and colouration at cooling (T_3_ and T_4_), respectively, for the colour hysteresis of the C6, pC6, and µC6 samples. See also Figs 1, S1 and S2.

Sample	Width (°C)	T_1_ (°C)	T_2_ (°C)	T_3_ (°C)	T_4_ (°C)
C6	—	−7	6	4	−6
pC6	4	−8	7	2	−12
µC6	18	−6	10	−6	−18

In the bulk state (C6), the composite discolours before the melting is completed (transition A) but it re-colours well above the freezing point (transition B). This confirms that colouration/discolouration of a TC composite may not follow the phase changes^15^, however, a coloured liquid is formed before solidification and colourless solid before melting. The corresponding colour hysteresis possesses an unusual shape where the cooling and heating curves intersect. This is in agreement with our previous research - TC composites have a very narrow colour hysteresis and in some cases the two curves may also cross each other^41^. When the composite is inside the paper (pC6), transitions A and B remain practically unaffected, but the colour hysteresis broadens to about 4 °C and becomes less steep than in the bulk. Moreover, discolouration of the sample at heating and re-colouration at cooling coincide well with the temperatures where melting (A) and crystallization (B) processes are completed. The µC6 sample shows two phase transitions at cooling, one at crystallization of its bulk form (B) and another one at much lower temperatures (C) and the colour hysteresis is very broad (∼18 °C). This sample discolours at higher temperatures than in the bulk form (C6) and inside the paper (pC6), but still at about 6 °C below the temperature at which the active core inside microcapsules completely melts. Re-colouration of the sample occurs at considerably lower temperatures than in the other two forms and completes just before the onset of the transition C.

Discolouration upon heating starts at virtually the same temperature in all three samples (T_1_) but completes at a higher temperature (T_2_) in the paper (pC6) and especially in the microcapsules (µC6). A stronger influence of the spatial conditions of the TC composite on its colouration and phase changes was recorded at cooling, the most pronounced in the microencapsulated form. A direct comparison of the thermal and colouration properties of the analysed TC composite is shown in Fig. S2 (Supplementary information).

### Thermal properties

**Figure 2:**
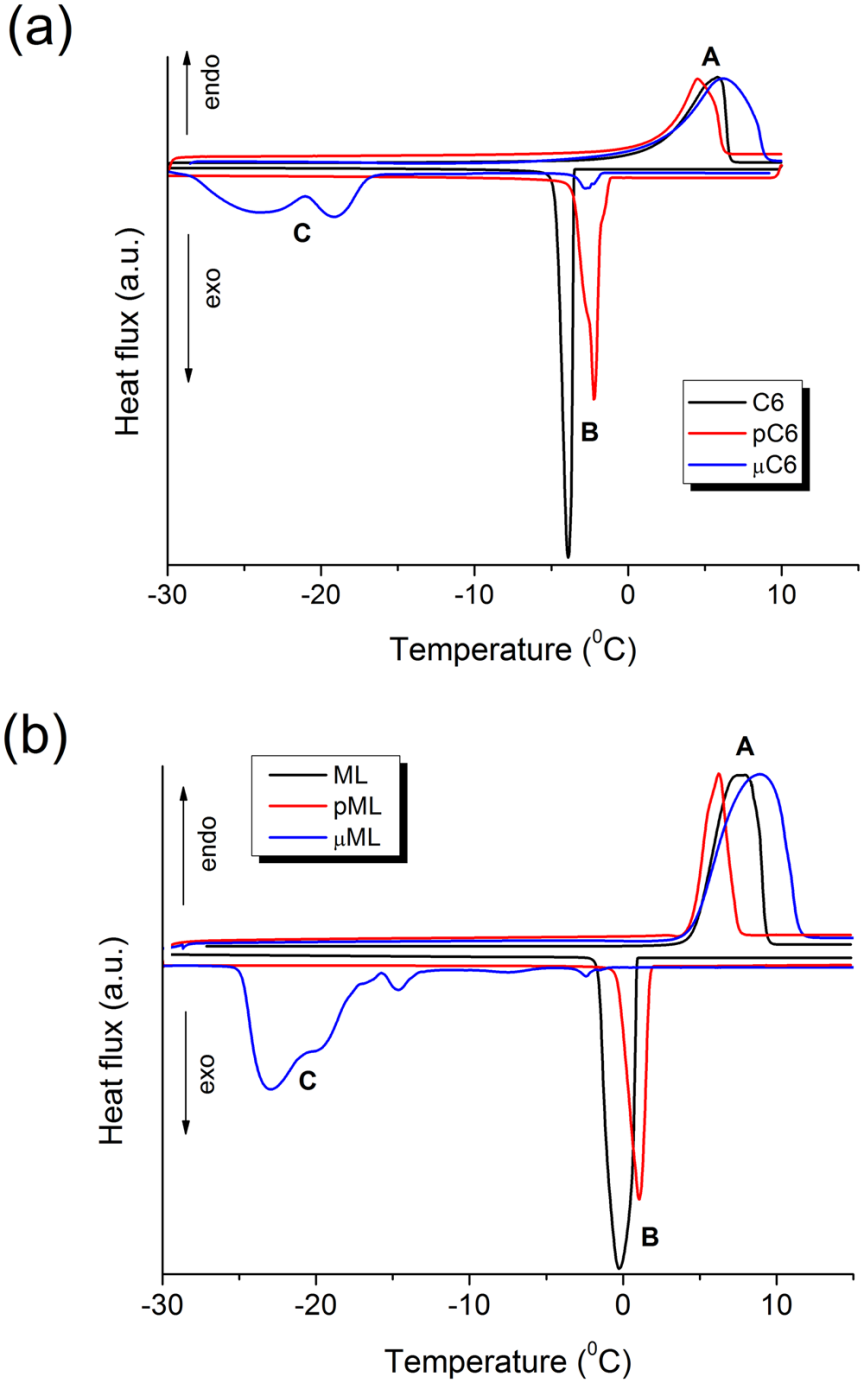
DSC thermograms of (**a**) the TC composite (C6, pC6, and µC6), and (**b**) the applied co-solvent (ML, pML, µML). The heat fluxes were normalized according to the melting peak (transition A). Both heating and cooling rates were 2 °C/min.

A slow DSC experiment (Fig. 2a) resolved the details obscured by the rapid scanning used to obtain the colorimetric measurements (Fig. 1). The results were compared with those for the ML co-solvent in the same three forms (Fig. 2b). The corresponding phase change temperatures and transition enthalpies are given in Table 2. The mass of the host material (paper and microcapsule’s shells) was not taken into account, therefore thermograms were normalized according to the melting peak (A) and transition enthalpies can be compared only among samples with the same holding material.

**Table 2:** The phase-change temperature (*T*_pc_) and the enthalpy (*ΔH*) of transitions A, B, and C of the analysed materials in bulk (C6, ML), inside the chromatographic paper (pC6, pML) and in microcapsules (µC6, µML).

Sample	Transition A		Transition B		Transition C	
	*T*_pc_ (°C)	*ΔH* (J/g)	*T*_pc_ (°C)	*ΔH* (J/g)	*T*_pc_ (°C)	*ΔH* (J/g)
C6	5.5	150	−2.4	−162	—	—
pC6	4.5	27	−2.0	−28	—	—
µC6	6.1	115	−2.2, −2.7	−5	−19.0, −24.0	−94
ML	8.0	530	1.0	−536	—	—
pML	6.3	33	0.6	−32	—	—
µML	8.7	135	−1.5, −2.4	−1	−14.6, −16.6, −20.0, −22.9	111

The temperatures of all resolved maxima are given for multiple peaks, while *ΔH* is for the entire transition. The mass of the paper and of the microcapsules’ shell was not taken into account, therefore only comparisons between samples with the same host material are reasonable.

DSC thermograms of the TC composite and of the applied co-solvent show the same features with systematically larger transition enthalpies with the latter, thus these transitions are driven by the co-solvent. Two (or even three) transition peaks were resolved at bulk crystallization (transition B), lower in µML and higher in µC6. The µML sample shows peaks also between transitions B and C where practically no features were observed in the µC6 sample (Fig. S3, Supplementary information).

The supercooling effect of transition B is very small, about 1 °C for the composite and about 2 °C for the co-solvent. Thus, the spatial separation of the PCM is not critical and transition B can be regarded as having a bulk nature. The size effect of the microcapsules is discussed later in the article.

To understand the measured crystallisation properties, two questions have to be answered – first, are the transitions B and C thermally related and, second, if the two transitions lead to the same crystal structure (i.e. to the same crystalline polymorph). The first question was addressed by combining static and dynamic DSC measurements and the second one by performing powder XRD measurements.

**Figure 3:**
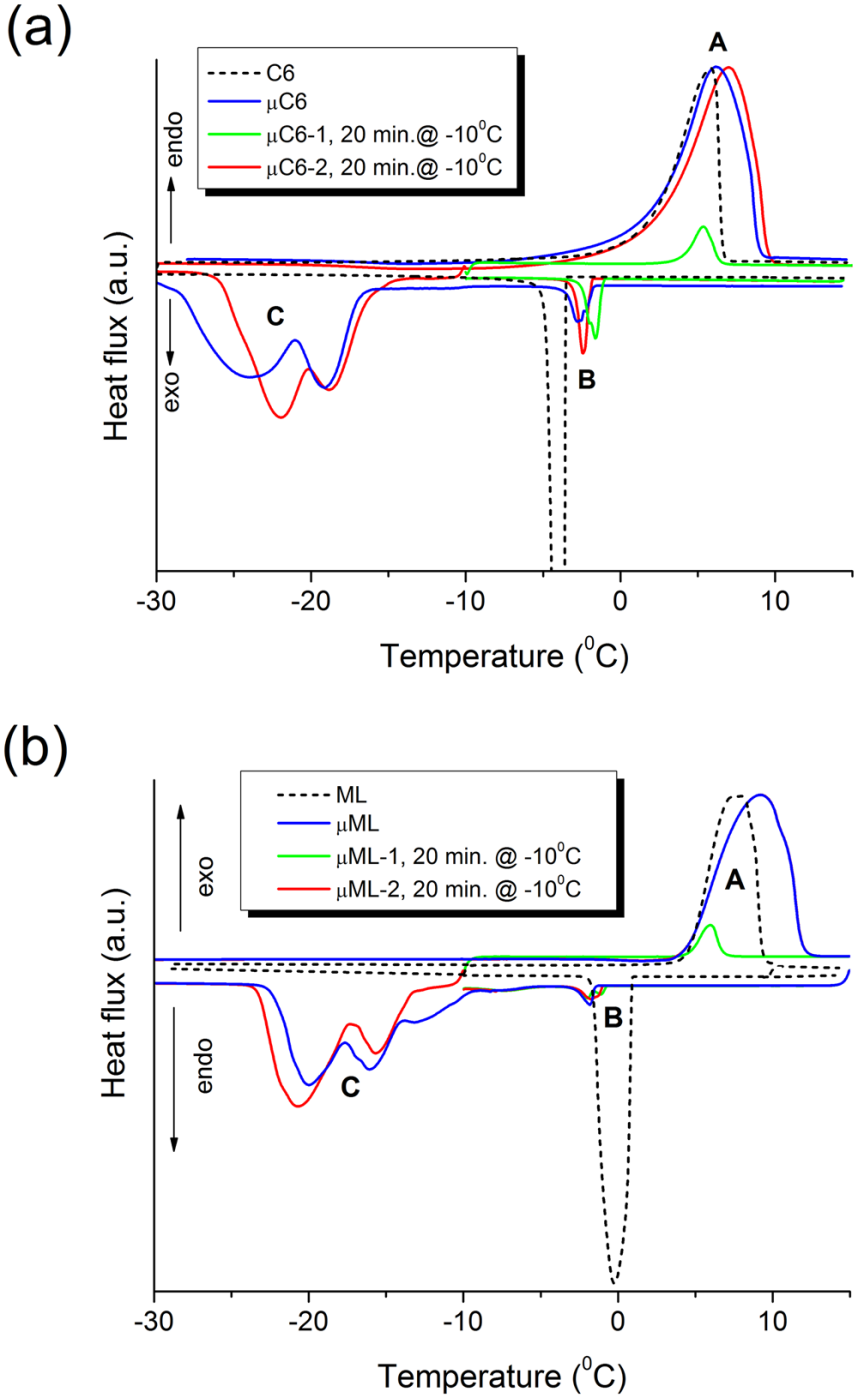
DSC thermograms of (**a**) the composite and (**b**) the co-solvent in the bulk (C6, ML) and microencapsulated forms (µC6, µML). Green curve: 20 min isothermal DSC was used after cooling to −10 °C, followed by heating. Blue curve: after 20 min isothermal DSC taken at −10 °C, the dynamic regime continued to −50 °C, followed by heating. Both heating and cooling rates in the dynamic regime were 2 °C/min.

The crystallization properties of the microencapsulated samples were analysed by applying a 20 minute isothermal regime between transitions B and C, i.e. at −10 °C (Fig. 3 and Table 3). When a 20 min isothermal DSC was used after cooling to −10 °C followed by the samples’ heating back to 15 °C, the melting enthalpy (transition A) was found to correspond reasonably well with the crystallization one (transition B). When after 20 min isothermal DSC at −10 °C the sample was cooled down to −50 °C, the peaks at transition C were similar to those when only a dynamic DSC was applied. Moreover, almost an identical curve was produced upon heating (compare blue and red curves in Fig. 3). The 20 minutes isothermal regime used at −10 °C caused a negligible crystallization, thus the transition A remained practically unaffected when cooling was stopped. Similar results were obtained for the TC composite and for the co-solvent. It is therefore safe to conclude that the transitions B and C are thermally independent.

**Table 3:** The phase change temperature (*T*_pc_) and the enthalpy (*ΔH*) of transitions A, B, and C measured for microencapsulated materials in dynamic regime over the entire temperature region (µML, µC6), using 20 min isothermal DSC at −10 °C in the −10 °C to 15 °C temperature region (µML-1, µC6-1) and in the entire temperature region with 20 min isothermal DSC at −10 °C (µML-2, µC6-2).

Sample	Transition A		Transition B		Transition C	
	*T*_pc_ (°C)	*ΔH* (J/g)	*T*_pc_ (°C)	*ΔH* (J/g)	*T*_pc_ (°C)	*ΔH* (J/g)
µML	9.0	142	−1,8	−3	−8.2, −13.1, −16.0, −19.9	−125
µML-1	6.0	8	−1.1, −1.9, −7.3	−4	—	—
µML-2	9.1	142	−1.7	−3	−15.6, −20.6	−121.0
µC6	6.1	116	−2.8	−5	−19.0, −23.9	−93.6
µC6-1	5.3	7	−1.6, −1.9	−6	—	—
µC6-2	6.9	124	−2.3	−6	−18.8, −21.9	−105

The temperatures of all resolved maxima are given for multiple peaks while *ΔH* is for the entire transition.

### Crystalline phase

**Figure 4:**
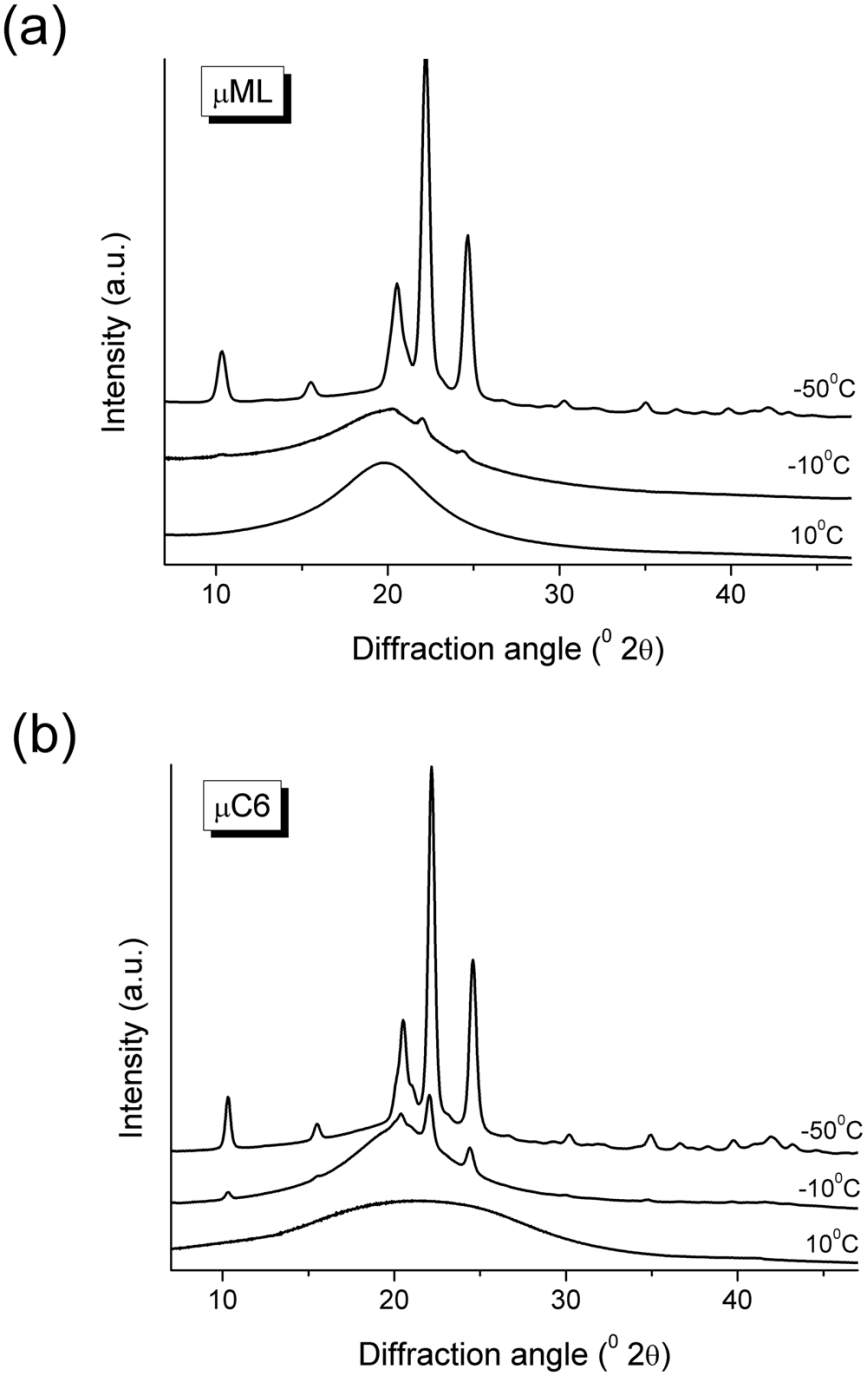
Powder XRD patterns of (**a**) µML and (**b**) µC6 samples after cooling to 10 °C, −10 °C, and −50 °C.

The crystalline phase, resulting from the transitions B and C was checked by powder XRD of the samples µML and µC6 cooled down to 10 °C, −10 °C and −50 °C. At 10 °C, both samples are amorphous, as expected – according to the DSC results, they are still liquid at this temperature. However, at −10 °C and −50 °C the diffraction patterns show the appearance of peaks, confirming that for both samples, the thermal effects are due to crystallization. The appearance of diffraction peaks at the same diffraction angles and having the same intensity ratios clearly indicates that the crystalline phase is apparently the same in all cases (both samples and both temperatures, just its amount is larger at −50 °C in both samples, see Fig. 4). We can conclude that the presence of the dye does not affect the crystallization to the extent that another polymorph of ML or co-crystal would crystallize. It has to be noted that no crystal structure data for ML are available so that structure-related discussion of the powder patterns is neither possible nor needed for the purpose of this work.

### Spatial confinement effect

**Figure 5:**
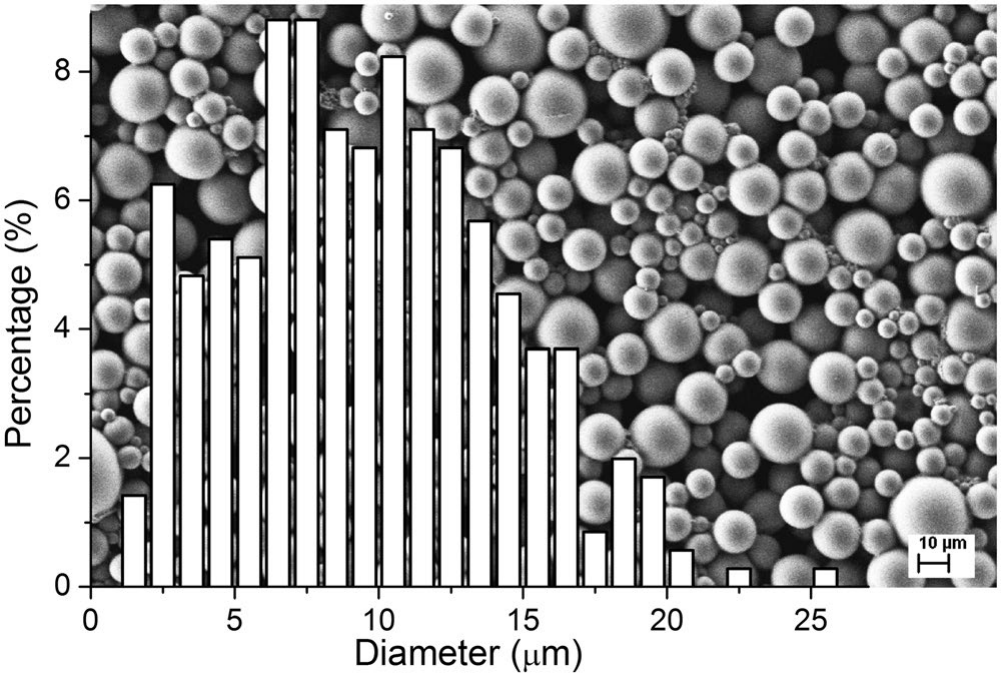
The size distribution and typical SEM micrograph of the µC6 sample.

The microencapsulated samples have a broad size distribution, ranging from 1.6 to 25.4 µm with the median 9.4 µm for µC6 (Fig. 5) and similar for µML (Fig. S4a). The samples have the same thickness as microcapsules’ shell (Table 4).

**Table 4:** Diameter (mean, median, and range of the size distribution) and shell thickness for the prepared microcapsules (µC6, µML) and for smaller (µC6-s, µML-s) and larger microcapsules (µC6-l, µML-l).

Sample	Diameter (µm)			Shell thickness (nm)
	Mean	Median	Range (min < > max)	
µC6	9.6	9.4	1.6 < > 25.4	119 ± 5
µC6-s	8.4	8.2	3.7 < > 15.3	
µC6-l	18.4	18.4	12.9 < > 25.1	
µML	9.8	9.2	1.6 < > 24.0	118 ± 5
µML-s	4.3	3.9	1.1 < > 11.3	
µML-l	16.8	16.5	9.6 < > 22.8	

The shell thickness was measured on the SEM micrographs where the capsules were cut with a scalpel.

**Figure 6:**
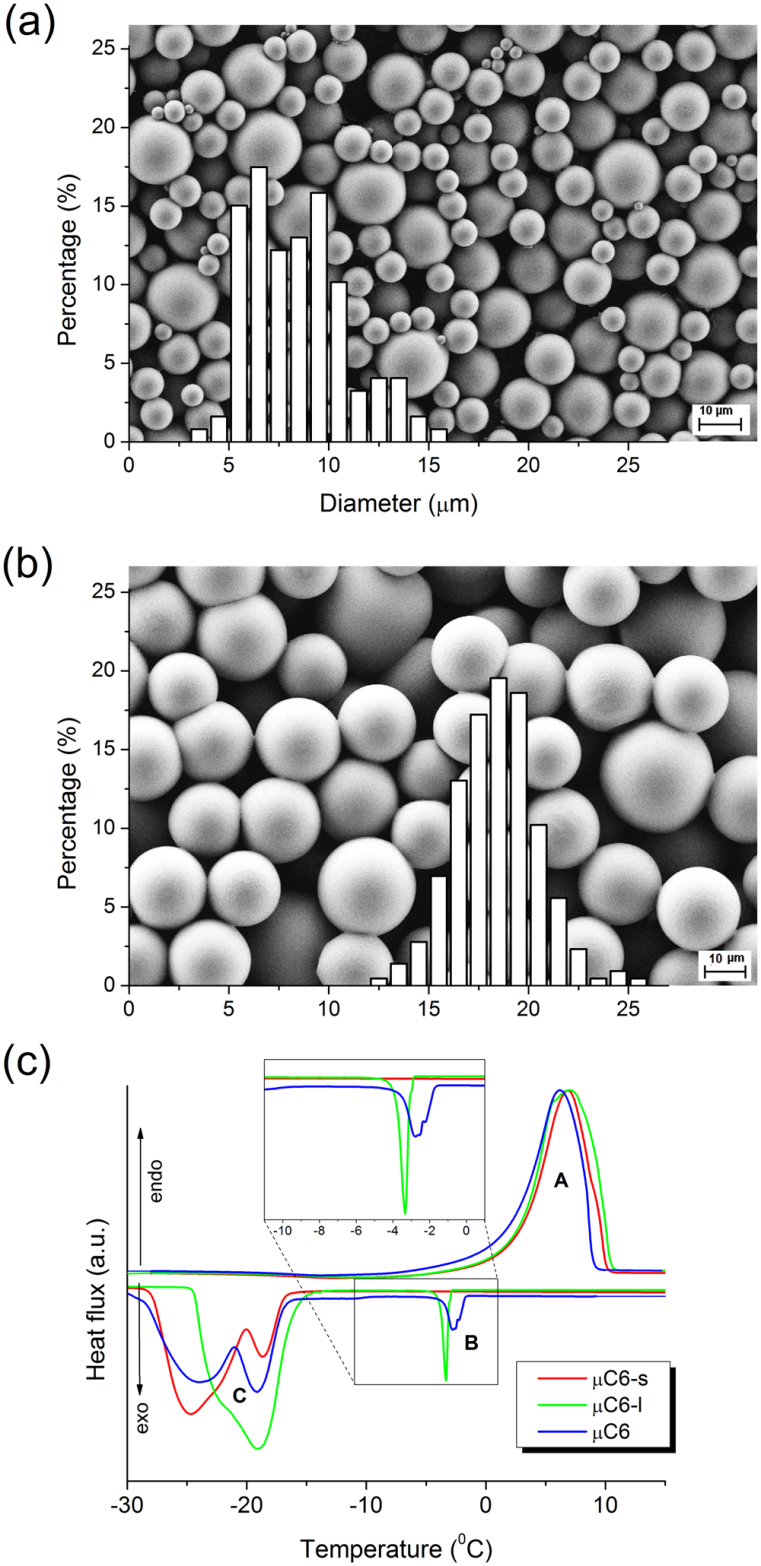
SEM micrographs of (**a**) µC6-s and (**b**) µC6-l samples with the corresponding particle size distributions. (**c**) DSC thermograms (2 °C/min) of µC6 (blue) and of its fractions µC6-s (red) and µC6-l (green), normalized according to the melting peak (transition A).

To investigate the influence of the microcapsules’ size on the colorimetric and thermal properties, the µC6 and µML dispersions were gravimetrically separated into fractions with smaller (µC6-s, µML-s) and larger microcapsules (µC6-l, µML-l), respectively; see Figs 6a,b, and S4a,b. The size of the microcapsules in the obtained samples is given in Table 4 and the corresponding DSC thermograms are shown in Figs 6c and S4d (Supplementary information) with the corresponding thermal data displayed in Table 5.

**Table 5:** The phase change temperature (*T*_pc_) and the enthalpy (*ΔH*) of transitions A, B, and C measured for microencapsulated ML and C6 materials in the as-prepared forms (µML, µC6) and after separation into samples with smaller (µML-s, µC6-s) and larger particle size (µML-l, µC6-l).

sample	Transition A		Transition B		Transition C	
	*T*_pc_ (°C)	*ΔH* (J/g)	*T*_pc_ (°C)	*ΔH* (J/g)	*T*_pc_ (°C)	*ΔH* (J/g)
µML	9.0	142	−1,8	−3	−8.2, −13.1, −16.0, −19.9	−125
µML-s	7.7	155	—	—	−14.5, −16.2, −19.7, −22.5	−134
µML-l	7.4	176	−1.9	−3	−14.4,−21.9	−141
µC6	6.1	115	−2.8	−5	−19.0, −23.9	−94
µC6-s	6.7	152	—	—	−18.6, −24.6	−139
µC6-l	6.8	165	−3.3	−7	−18.9, −22.0	−140

The temperatures of all resolved maxima are given for multiple peaks while *ΔH* is for the entire transition.

The transition B does not appear in DSC thermograms for smaller microcapsules (<11 µm, µC6-s and µML-s), but only for larger ones. The transition C consists of multiple peaks (Figs 6c, S4), which is typical for crystallization in mini-emulsion droplets^36–38^.

Thermal properties of the TC composite and of the applied co-solvent are very similar, leading to the conclusion that the phase changes of the TC composite are predominantly driven by the co-solvent while the other two components (leuco dye and developer) have only a minor influence. However, the colouration-discolouration ability is subject to the dye-developer interactions in the phase-changing co-solvent, and discolouration requires the interaction between a developer and a co-solvent. This interaction might strongly depend on the host material, especially on its morphology and the latter can be of considerable importance. For a deeper understanding of this topic, spectroscopic investigations would be necessary; however, the contribution of the host material will inevitably obscure the properties of the composite. In addition, good temperature stability during measurements must be assured. We will try to do some progress in this direction in our future work.

## Conclusions

Colour changes of the analysed leuco dye-based TC composite become loosely related to its phase transitions when the material is restricted within micrometer dimensions of a holding medium. The strongest effects are observed in microcapsules when cooling where their colour changes well before crystallization and the corresponding hysteresis is extremely wide. Two thermally independent exothermic transitions with the same crystalline structure were found – a weak one at bulk crystallization temperature and a much stronger one at a more than 15 °C lower temperature. Apparently, the low-temperature crystallization shifts the colouration of the microencapsulated composite to much lower temperatures, but not all the way to the phase change temperature. On the other hand, the open nature of the porous structure with narrower particle sizes than the smallest microcapsules shows only the bulk crystallization and the colour changes follow the phase transitions reasonably well. Here, the colour hysteresis exhibits a standard loop shape.

To the best of our knowledge, this is the first report of the influence of spatial confinement on the colour- and phase changes of a leuco dye-based TC composite. Both phenomena depend on the conditions in which the PCM is captured, but are only loosely related to each other. The results presented in the paper are important for designing different applications where such materials are used.

## Methods

Crystal violet lactone leuco dye (CVL, >95%, Tokyo Chemical Industry), benzyl 4-hydroxybenzoate (B4HB, >99%, Tokyo Chemical Industry) and methyl laurate (ML, 96%, Acros OrganicsTM, Fisher Scientific) were used as received. The TC composite (C6) was prepared by dissolving B4HB and CVL in the ML co-solvent in molar ratio CVL:B4HB:ML = 0.5:6:100.

The chromatography paper No. 232-674-98 (85 g/m², 2-4 µm pore size, Machery-Nagel GmbH & Co. KG) was used to spread the material into the porous paper (pC6, pML).

Microencapsulation (µC6, µML) was done by *in situ* polymerization of amino-aldehyde prepolymers^42,43^. All materials for the microcapsule shell originated from the continuous aqueous phase of the oil-in water emulsion. A modifying agent was added to enable polymerization of the aminoaldehyde precondensate only at the surface of the emulsified microcapsule cores. Under controlled polymerization conditions (pH, temperature) total mass of the shell material precipitated and was distributed evenly over the surface of droplets in the emulsion. This resulted in a uniform microcapsule shell thickness, regardless of the microcapsule size. Such result was also reported in previous studies^44,45^.

The size distribution of microcapsules was obtained by image analysis of SEM micrographs (Karl Zeiss Supra 35 C) using ImageJ image processing software with suitable numerical procedures^46^.

Temperature-dependent colour of the C6 was measured inside a cylindrical groove (11.2 mm diameter, 0.3 mm depth) in the 2.9 mm thick white-coated copper plate. The colour of the µC6 was measured on a thin layer of microcapsules prepared over a white paper using the Film-Applicator (Byk Additives&Instruments) with 200 µm clearance gap and dried at room temperature. All samples were cooled by dry ice (−78.5 °C) down to −30 °C and heated on a laboratory table at room temperature, producing cooling- and heating rates of about 10 °C/min and 5 °C/min, respectively. Temperature of the sample surface was measured by Ni Cr-Ni thermocouple connected to IR thermometer (Fluke 561). Colour of the samples was measured by the i1 Pro spectrometer (X-Rite, USA). The CIELAB colour values with D50 illuminant and 2° standard colorimetric observer were calculated by KeyWizzard software^47^. The colour change was expressed by the CIE colour difference ΔE_ab_* with respect to the highest measured temperature and normalized according to the totally coloured state taken at the lowest temperature.

Thermal properties were analysed by differential scanning calorimetry (DSC) using Mettler Toledo DSC1 apparatus at heating/cooling rates of 2 °C/min, 5 °C/min, and 10 °C/min from −50 °C to 15 °C. For this purpose, the water dispersions of microcapsules were dried at room temperature.

The XRD patterns were collected on the Rigaku SuperNova diffractometer, equipped with Atlas CCD detector, using CuKα radiation and the Powder power tool within the CrysalisPro data collection software (version 171.37.34t). The cooling of the samples was done using an Oxford Instruments CryoJet device.

## Electronic supplementary material

Supplementary Information
