# Supplementary material for: Relation between colour- and phase changes of a leuco dye-based thermochromic composite
Source: Sci Rep. 2018 Apr 3;8:5511. doi: 10.1038/s41598-018-23789-2 (PMC5882991; doi:10.1038/s41598-018-23789-2)
Supplement: Supplementary file 1 — Supplementary Information [file 41598_2018_23789_MOESM1_ESM.pdf]

Supplementary information

Relation between colour- and phase changes of a leuco dye-based thermochromic composite

***Kristina Bašnec*[1], *Lidija Slemenik Perše*[2], *Boštjan Šumiga*[3], *Miroslav Huskič*[2], *Anton Meden*[4], *Aleš Hladnik*[3], *Bojana Boh Podgornik*[3], *and Marta Klanjšek Gunde*[2*]**

[1]*Radeče Papir Nova, d.o.o., Njivice 7, SI-1433 Radeče, Slovenia*

[2]*National Institute of Chemistry, Hajdrihova 19, SI-1000 Ljubljana, Slovenia*

[3]*University of Ljubljana, Faculty of Natural Sciences and Engineering, Aškerčeva cesta 12, SI-1000 Ljubljana, Slovenia*

[4]*University of Ljubljana, Faculty of Chemistry and Chemical Technology, Večna pot 113, SI-1000 Ljubljana, Slovenia*

** Corresponding author:*
Tel: +3861 4760291; fax +3861 4760300,
e-mail: marta.k.gunde@ki.si (M. Klanjšek Gunde)

Keywords: thermochromic materials; colour changes; phase changes; phase change materials; microencapsulated material; spatial confinement; methyl laurate.

Supplementary information 1

Description of colour properties

Temperature-dependent colour of a typical leuco dye-based thermochromic compound displays a hysteresis. Its shape was described by the width of the loop and the four characteristic temperatures, describing the onset (T1 and T3) and termination (T2 and T4) of the TC effect: discoloration at heating (T1 and T2) and colouration at cooling (T3 and T4), respectively (Figure S1). The temperatures where the colour difference between the two adjacent measurements exceeds 2 CIELAB units were used for this purpose, which is about 3% of the entire colour change and is comparable to the experimental error. The width of each loop was determined at the half-height of the hysteresis.

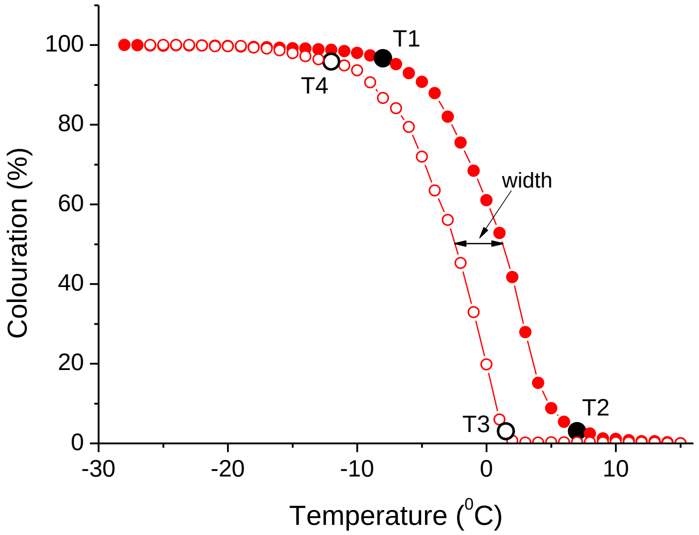

Figure S1. The characteristic temperatures and the width of a typical colour hysteresis. The colour obtained at heating is represented by filled circles and those at cooling by empty ones. The shown hysteresis was measured for the pC6 sample (TC composite inside chromatographic paper).

Supplementary information 2

Comparison of thermal and colour properties of the TC composite in three forms

DSC thermograms and colour changes as a function of temperature were measured by the same heating and cooling rates to enable direct comparison of the two properties for the same TC composite in three forms: in bulk, in a porous medium (chromatographic paper) and in microcapsules.

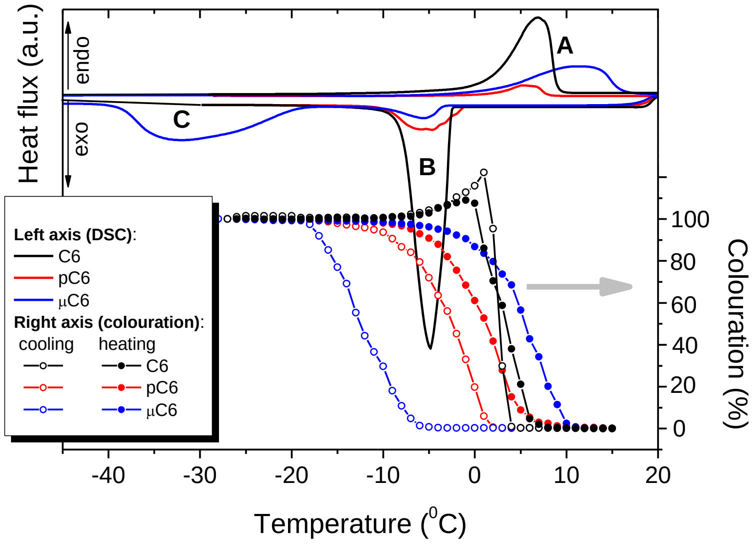

Figure S2. Thermal (DSC) and colorimetric properties of the TC composite in bulk (C6), inside chromatographic paper (pC6) and in microcapsules (µC6). Heating and cooling rates of 5 °C/min and 10 °C/min, respectively, were used for both measurements.

Supplementary information 3

DSC thermograms of microencapsulated TC composite and the used co-solvent

Transition temperatures and enthalpies are displayed in the article in Table 2.

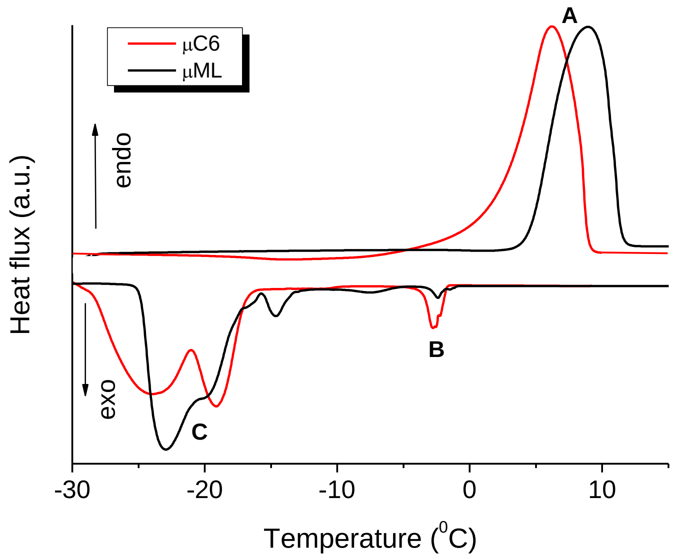

Figure S3. DSC thermograms of the microencapsulated composite (μC) and of the microencapsulated co-solvent (μML). The heat fluxes were normalized according to the melting peak (transition A). Heating and cooling rate of 2 °C/min was used.

Microencapsulated co-solvent (μML) produces a larger number of crystallization peaks compared to the composite (μC6). This might be due to a higher number of nucleation sites available in the μC6, resulting in a less probable homogeneous nucleation.

Supplementary information 4

The influence of microencapsulation on the thermal properties of the used co-solvent

SEM pictures of the microencapsulated co-solvent were used to determine the particle size distribution. The procedure was accomplished for the entire sample (µML) and for both fractions, made by gravimetric separation, µML-s and µML-l.

DSC graphs were measured for the three samples, to analyse the properties on cooling.

(a)

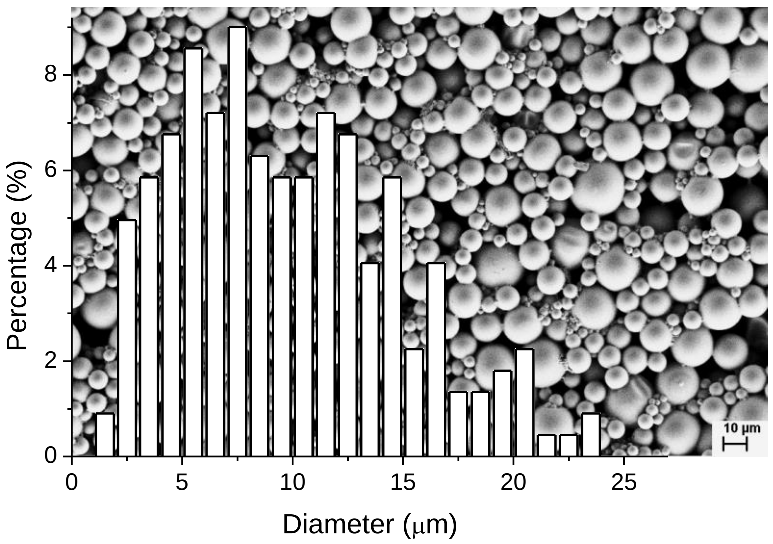

(b)

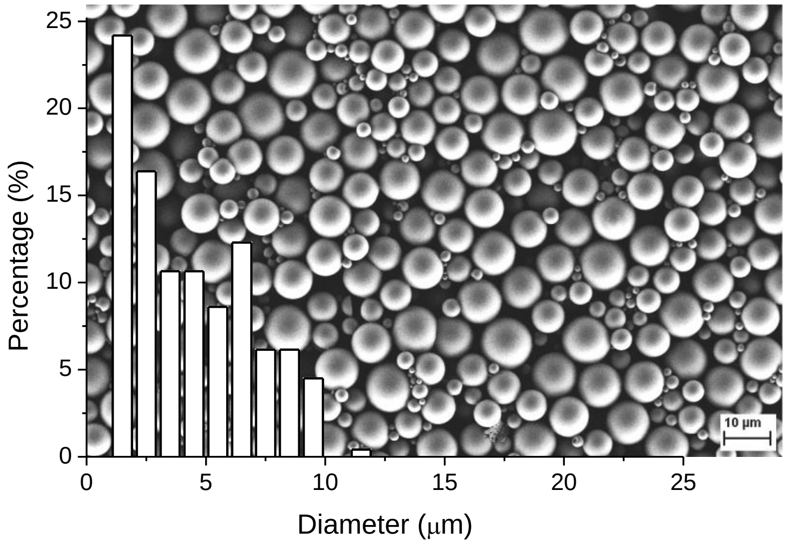

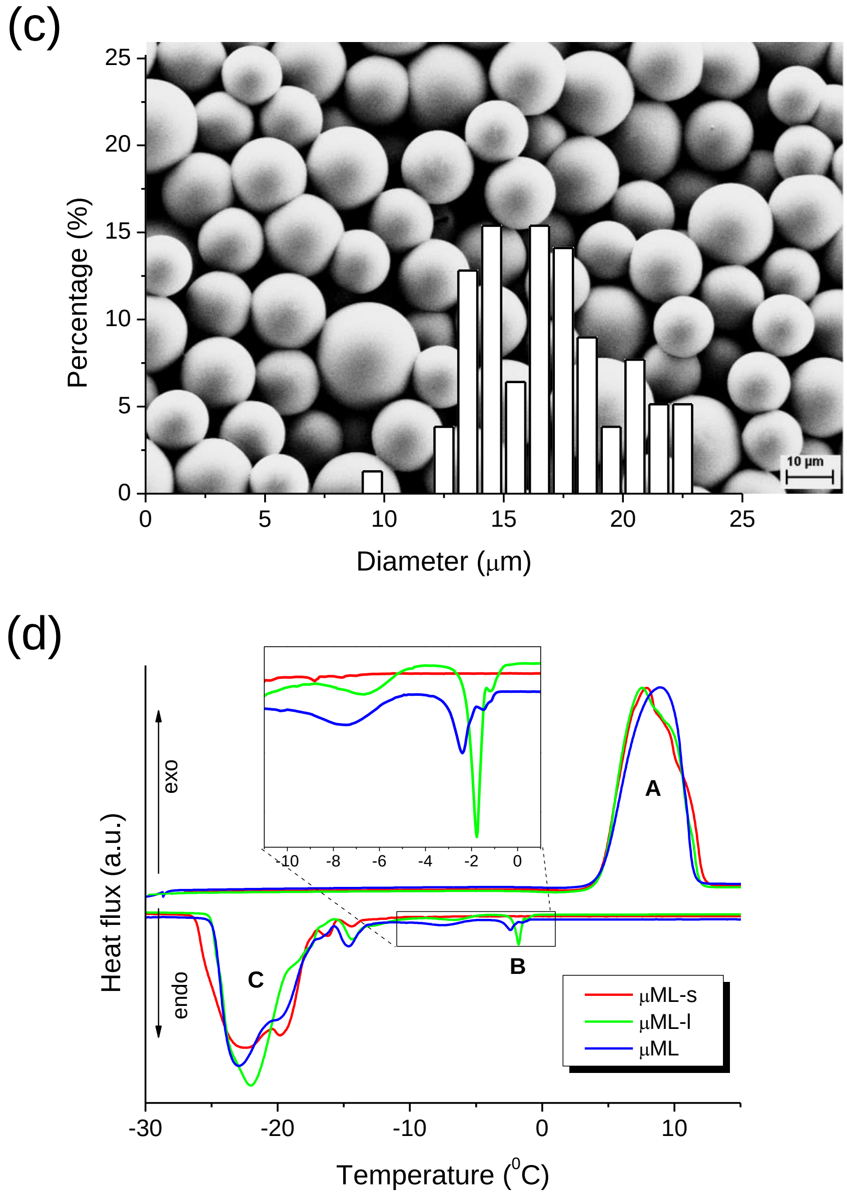

Figure S4. SEM micrographs of (a) μML (entire sample), (b) μML-s (smaller capsules), (c) μML-l (larger capsules) samples with the corresponding particle size distributions. (d) DSC thermograms (2 °C/min) of μML (blue) and of its fractions μML-s (red) and μML-l (green), normalized according to the melting peak (transition A).
